# Place of food cooking is associated with acute respiratory infection among under-five children in Ethiopia: multilevel analysis of 2005–2016 Ethiopian Demographic Health Survey data

**DOI:** 10.1186/s41182-020-00283-y

**Published:** 2020-11-30

**Authors:** Abraham Geremew, Selamawit Gebremedhin, Yohannes Mulugeta, Tesfaye Assebe Yadeta

**Affiliations:** 1 Department of Environmental Health, College of Health and Medical Sciences, Haramaya University, Dire Dawa, Ethiopia; 2 School of Nursing and Midwifery, College of Health and Medical Sciences, Haramaya University, Dire Dawa, Ethiopia

**Keywords:** Acute respiratory infection, Food cooking place, Solid biomass fuel

## Abstract

**Background:**

Globally, acute respiratory infections are among the leading causes of under-five child mortality, especially in lower-income countries; it is associated with indoor exposure to toxic pollutants from solid biomass fuel. In Ethiopia, 90% of the population utilizes solid biomass fuel; respiratory illness is a leading health problem. However, there is a paucity of nationally representative data on the association of household cooking place and respiratory infections. Besides, evidence on the variability in the infection based on the data collected at different times is limited. Therefore, this study is intended to assess the association of food cooking place with acute respiratory infections and the variability in households and surveys.

**Methods:**

The current analysis is based on the Ethiopian Demographic and Health Survey data collected in 2005, 2011, and 2016 and obtained via online registration. The association of food cooking place with acute respiratory infection was assessed using multilevel modeling after categorizing all factors into child level and survey level, controlling them in a full model. The analyses accounted for a complex survey design using a Stata command “svy.”

**Result:**

A total of 30,895 under-five children were included in this study, of which 3677 (11.9%) children had an acute respiratory infection, with 12.7% in 2005, 11.9% in 2011, and 11.1% in 2016. The risk of having an infection in under-five children in households that cooked food outdoors was 44% lower (AOR = 0.56, 95% CI = 0.40, 0.75) compared to those households that cooked the food inside the house. There was a statistically significant difference among the children among surveys to have an acute respiratory infection.

**Conclusion:**

The risk of having children with acute respiratory infection is lower in the households of cooking food outdoor compared to indoor. The infection difference in different surveys suggests progress in the practices in either food cooking places or the fuel types used that minimize food cooking places location or the fuel types used that minimizes the risk. But, the infection is still high; therefore, measures promoting indoor cooking in a well-ventilated environment with alternative energy sources should take place.

## Background

Acute respiratory infection (ARI) is a leading cause of mortality in children under 5 years old worldwide [1]. About 97% of ARI cases occur in low- and middle-income countries, with nearly 70% occurring in South Asia and sub-Saharan Africa [2]. The World Health Organization report in 2015 shows that ARI accounts for 16% of all deaths globally, killing around one million children under the age of 5 years [3]. In 2017, of nearly 5.4 million under-five children who died worldwide, roughly half were in sub-Saharan Africa, with ARI contributing the highest number of deaths [4]. The recent analysis of the demographic and health survey data (DHS) in sub-Saharan countries shows that the overall prevalence is 25.3% [5].

Multiple risk factors are associated with ARI including exposure to toxic pollutants in the indoor environment, biomass smoke being one of the most common ones [6–10]. Smoke from biomass and coal contains a large number of particulate matters of various sizes such as nitrogen dioxide, carbon monoxide, methylene chloride, and dioxins [7, 8, 10]. Women and under-five children are exposed to these pollutants because of the long periods of time spent in close proximity to fires while cooking and heating [11]. Pollutants generated in kitchens and heating areas can also spread into living areas where children and other household members are exposed [11, 12]. Globally, despite the health risks of the use of biomass fuels such as animal dung, crop residues, wood, and coal for cooking and heating purposes continues, approximately 3 billion people continue to use these various biomass fuels worldwide [13, 14]. The recent analysis of demographic and health data in Afghanistan illustrated that in addition to using such fuels, the location used to cook the food had a significant association with acute respiratory infections [15]. The evidence indicates controlling the exposure to biomass fuels could reduce the risk of adverse children and adult health outcomes by 20–50% [16]. Outdoor cooking and stove ventilation were some of the measures that substantially reduced the risks [15, 17, 18].

In Ethiopia, there are 55 deaths per 1000 live births in children under-five due to acute respiratory infection, placing it among the world’s highest mortality rates [19]. Prior studies indicated that maternal age, residence, maternal hand hygiene information [20], maternal literacy, smoking, use of animal dung as a fuel source, nutritional status [21], absence of a separate kitchen, and lack of windows were significantly associated with acute respiratory infections [22–25].

In Ethiopia, more than 90% of the population uses solid biomass fuel [26–28]; there is a paucity of evidence on the location of cooking food and the association with ARI in children under-five, based on a nationally representative community-based study. Moreover, there is limited evidence on whether there is variability in the infection over time. Therefore, the current study is intended to assess the association of the location of cooking food with acute respiratory infection in under-five children and its variability in households and surveys based on demographic and health survey data collected in three different time periods. The results will provide evidence for policymakers for better intervention in progressing the under-five mortality and morbidity of the SDG strategic plan.

## Methods

### Study setting

According to the world population review report in 2020, Ethiopia has an estimated 114.96 million population, making it the second-largest country in Africa, the twelfth most populous country worldwide [29]. Ethiopia has a population growth rate of 3.02% per year and a fertility rate of 4.73% [29, 30]. The administrative structure consists of nine regional states (Tigray, Afar, Amhara, Oromiya, Somali, Benishangul-Gumuz, Southern Nations Nationalities and People (SNNP), Gambela, and Harari) and two city administrations (Addis Ababa and Dire Dawa) [31].

### Study design, sample size, and sampling procedure

The Ethiopian Demographic and Health survey 2005, 2011, and 2016 were used for the current study. The surveys were conducted among women aged 15–49 years from urban and rural areas in the nine regions and two cities. During the surveys, each of the regions was stratified into urban and rural areas. Samples of Enumeration Areas (EAs) were selected independently from each stratum from where the samples were determined using a stratified, two-stage cluster design. The number of EAs from the 2016 survey was 645 (202 from urban and 443 from rural); for the 2011 survey, 624 (187 from urban and 437 from rural areas); for the 2005 survey, and 540 (145 from urban and 395 from rural areas) [26–28]. The number of households and clusters included was 14,500 from 540 clusters in 2005; 17,817 from 624 clusters in 2011; and 16,650 from 645 clusters in 2016. The sampling procedure applied is as follows: first, clusters were selected from the enumeration area list from the population and housing census sample frame. The sampling frame for the 2005 survey was based on the 1997 census; the 2011 and 2016 surveys were based on the 2007 census. Secondly, the households were selected after listing the households in all selected enumeration areas for the sampling frame [26–28]. Based on the data obtained, the number of children included in the three surveys was 30,985 (9517 from the 2005 survey, 11,176 from the 2011 survey, and 10,291 from the 2016 survey).

### Method of data collection

The data collection method was face-to-face interviewing of mothers or respondents, as indicated elsewhere [26–28].

### Outcome variable

The outcome of interest was acute respiratory infection (ARI). The DHS survey assessed whether the children of participating mothers suffered from a cough in the last 2 weeks before the survey. The respondents were further probed whether the cough was associated with shortness of breath or rapid breathing problems during the specified period. Therefore, children who suffered a cough with shortness of breathing were considered to have ARI.

During each survey, households were asked about the type of fuels they reportedly used and the place of cooking food. Respondents were categorized into solid biomass fuel users (families that used wood, charcoal, kerosene, grass, crop products) or non-solid biomass fuel users (electricity, liquid petroleum gas, natural gas, biogas). Besides, households were asked about the location where they cooked the food (either inside the house, outside, or in a separate building).

### Independent variables

Variables were selected based on prior findings that revealed their association with children’s ARI [5, 15, 23, 25, 32–36]. These were the child’s age, place of cooking food, maternal age, maternal education, paternal education, residency, nutrition status, household wealth quintile, frequency of watching television, frequency of listening to radio, maternal occupation, and paternal occupation, as indicated elsewhere. Stunting, underweight, and wasting was assessed based on the World Health Organization Child Growth Standards and the National Center for Health Statistics (NCHS)/WHO international growth reference [37].

Variables were classified into three levels: (1) child-level variables (age of the child, sex of the child, size of the child, child weight, child stunting, child wastage, and breastfeeding), (2) household-level variables (wealth quintiles, water sources, toilet type, residence, maternal education, maternal occupation, paternal education, paternal occupation, frequency of listening radio, and frequency of watching television), and (3) survey-level variables (the type of fuel, location of cooking food). However, the type of fuel was excluded from analysis as more than 98% of households were reportedly using solid biomass fuel.

### Data analysis

Data were analyzed using the Stata 14 version (Stata Corp LP, College Station, TX, USA). The “svy” command was used to weigh the survey data to adjust cluster sampling design in the merged data set of three surveys. First, the study population’s characteristics were presented by ARI status and by exposure variables. The association of ARI with the place of cooking food was examined using a three-level multilevel logistic regression to assess variability in the outcome using a household and survey as level. A multilevel analysis was used due to the level of observation at which the child’s ARI status is nested within households and surveys. Inclusion of the higher-level (household and survey-level) characteristics as child-level factors can lead to the understatement of standard error, as one value was replicated across all members of the same group. Using a multilevel model, the value was applied once, at the group level, and information from the pooled regression helped generate reliable estimates even for groups with insufficient numbers of first-level observations. The model also allowed to include error terms at each level, tracking changes in variance across models [38, 39]. The model is based on the following equation:

1 $$ {\mathrm{Y}}_{\mathrm{i}}={\mathrm{a}}_{\mathrm{jk}}+{\upbeta \mathrm{X}}_{\mathrm{i}}+{\mathrm{e}}_{\mathrm{i}}\kern0.6em i=1,\hbox{-} \hbox{-} \hbox{-} \hbox{-} \hbox{-} \hbox{-} \hbox{-} \hbox{-} \hbox{-} \hbox{-} \hbox{-} \hbox{-} \hbox{-}, \mathrm{I} $$

2 $$ {\mathrm{a}}_{\mathrm{j}\mathrm{k}}={\mathrm{g}}_0^{\mathrm{j}}+{\mathrm{g}}_{\mathrm{k}}+{\mathrm{e}}_{\mathrm{j}}\kern0.48em \mathrm{j}=1,\hbox{-} \hbox{-} \hbox{-} \hbox{-} \hbox{-} \hbox{-} \hbox{-} \hbox{-} \hbox{-} \hbox{-} \hbox{-} \hbox{-} \hbox{-}, \mathrm{J}\kern0.73em \mathrm{k}=1\hbox{-} \hbox{-} \hbox{-} \hbox{-} \hbox{-} \hbox{-} \hbox{-} \hbox{-} \hbox{-} \hbox{-} \hbox{-}, \mathrm{K} $$

3 $$ {\mathrm{g}}_{\mathrm{k}}={\mathrm{l}}_0+{\mathrm{l}}_{\mathrm{k}}+{\mathrm{D}}_{\mathrm{k}}+{\mathrm{e}}_{\mathrm{i}}\kern0.48em \mathrm{k}=1,\hbox{-} \hbox{-} \hbox{-} \hbox{-} \hbox{-} \hbox{-} \hbox{-} \hbox{-} \hbox{-} \hbox{-} \hbox{-} \hbox{-} \hbox{-} \hbox{-}, \mathrm{K} $$

where *Y*_i_ the binary response of mother on child i about ARI status in the *j* household in *k* survey, ***a***_jk_ and *β* are the intercept and coefficient vector for child-level variables *X*_i,_ *g*^j^_0_ is a household-specific intercept, and *g*_k_ survey-level intercept which is a function of survey-level variable *D*_k,_ survey-level coefficient *l*_k_, and survey-level intercept *l*_0_. Finally, *e*_i_, *e*_j_, and *e*_k_ are error terms at each level.

### Model building

Before the model building, a binary multilevel logistic regression was applied. Those variables with a *p* value of less than 0.2 were considered for the multivariable analysis to estimate the adjusted odds ratios and the extent of random variations between households and surveys. Three models containing variables of interest was fitted using the “svy: xtmelogit” command in Stata: model-I (empty model): model fitted without factors to test the random variability in the intercept, model-II: to assess the effect of the survey-level variable and (random intercept and slope for survey-level variables), and model-III (full model): to evaluate the impact of survey-level variables and other variables and simultaneously. In a full model, the child-related variables included were age, sex, birth weight, and nutrition status and the household-related variables were wealth quintiles, listening radio, watching television, drinking water source, toilet type, maternal age, maternal education, paternal education, paternal occupation, maternal occupation, and residence. All statistical tests were considered significant at *p* value < 0.05.

### Ethical consideration

The three EDHS were conducted after obtaining ethical approval from the ICF Institutional Review Board (IRB), Ethiopia Health and Nutrition Research Institute Review Board, and the Ministry of Science and Technology. The data collectors were instructed in how to obtain informed consent statement and voluntary participation prior to data collection. The confidentiality of the information was maintained. For this particular study, a brief description of the protocol was submitted to the MEASURE DHS program to access and analyze the data.

## Result

### Socio-demographic characteristics of households with under-five children

Nine thousand nine hundred seventy-one children were included in the analysis from the 2005 survey, 10729 from the 2011 survey, and 10195 from the 2016 survey, with a total of 30895. Two-fifths of the children had an average size, and nearly one-fifth of the children had an age of 3 years and 4 years each. In total, nearly nine-tenths of the children reside in rural, 42% of the children were stunted, 30% were underweight, and 17% were wasted. The majority of participants were from rural areas, and about two-fifths were stunted. Stunting decreased over time, with 50% stunting in 2005, 42% in 2011, and 38% in 2016. Similarly, the number of underweight children also decreased from 50% (2005), 27% (2011), and 24% in 2016. Households’ experience of listening to the radio twice a week in 2005 was nearly 25% which increased to 32% in 2011 and dropped to 13% in 2016. On the other hand, household television watching increased to three times a week in 0.7% in 2005 to 8.3% in 2011 and 8.1% in 2016. Of mothers included in the survey, the majority was unemployed and more than 70% did not attend school (Table 1).

**Table 1:** Socio-demographics of households and under-five children characteristics, EDHS 2005–2016

Characteristics	Survey year, *N* (%)			Total
	2005	2011	2016	*N* (%)
Residence				
Urban	762 (7.0)	1427 (12.5)	1149 (10.7)	3338 (10.1)
Rural	10,075 (93.0)	9982 (87.5)	9559 (89.3)	29616 (89.9)
Stunting				
Normal	2222 (50.2)	6210 (58.6)	5987 (61.9)	14420 (58.4)
Stunted	2208 (49.8)	4397 (41.5)	3679 (38.1)	10283 (41.6)
Weight				
Normal	2222 (50.2)	7747 (73.0)	7368 (76.0)	17337 (70.1)
Underweight	2208 (49.8)	2860 (27.0)	2327 (24.0)	7395 (29.9)
Wasting				
Normal	2222 (50.2)	9637 (90.9)	8710 (89.9)	20569 (83.2)
Wasted	2208 (49.8)	971 (9.2)	978 (10.1)	4156 (16.8)
Breast feeding				
Never	380 (3.5)	332 (2.9)	520 (4.9)	1231 (3.8)
Ever	10,302 (96.5)	11,028 (97.1)	10,189 (95.2)	31519 (96.2)
Child age in years				
0 years	2211 (22.0)	2324 (21.5)	2243 (21.9)	6778 (21.8)
1 years	1869 (18.6)	1878 (17.4)	1966 (19.2)	5713 (18.4)
2 years	1875 (18.7)	2016 (18.7)	1911 (18.7)	5803 (18.7)
3 years	2081 (20.7)	2342 (21.7)	1973 (19.3)	6396 (20.6)
4 years	2000 (19.9)	2229 (20.7)	2150 (21.0)	6379 (20.5)
Size of child				
Very small	2411 (22.3)	2191 (19.3)	1890 (17.8)	6492 (19.8)
Smaller than average	1013 (9.4)	1451 (12.8)	1491 (14.0)	3954 (12.1)
Average	4343 (40.2)	4365 (38.4)	4457 (42.0)	13165 (40.2)
Larger than average	792 (7.3)	1005 (8.8)	1072 (10.1)	2868 (8.8)
Very large	2237 (20.7)	2357 (20.7)	1712 (16.1)	6307 (19.2)
Education of father				
No education	6322 (58.9)	5651 (50.2)	4866 (48.2)	16839 (52.5)
Primary	3256 (30.4)	4722 (42.0)	4028 (39.9)	12006 (37.4)
Secondary	1041 (9.7)	530 (4.7)	771 (7.6)	2342 (7.3)
Higher	109 (1.0)	351 (3.1)	438 (4.3)	898 (2.8)
Occupation of father				
Did Not Work	46 (0.43)	93 (0.8)	781 (7.7)	920 (2.9)
Professional	170 (1.6)	325 (2.9)	402 (4.0)	896 (2.8)
Sales	547 (5.1)	867 (7.7)	677 (6.7)	2091 (6.6)
Agriculture	9468 (88.3)	9057 (80.3)	6719 (66.4)	25244 (79.4)
Service	60 (0.6)	162 (1.4)	267 (2.6)	489 (1.7)
Skilled	245 (2.3)	591 (5.2)	549 (5.4)	1385 (4.4)
Unskilled	178 (1.7)	116 (1.0)	364 (3.6)	658 (2.1)
Other	15 (0.1)	69 (0.6)	359 (3.6)	443 (1.4)
Occupation of mother				
Did not work	7671 (70.9)	5306 (46.9)	5975 (55.8)	18953 (57.5)
Sales	764 (7.1)	1860 (16.5)	1234 (11.5)	3858 (11.7)
Agriculture	2042 (18.9)	3106 (27.5)	2398 (22.4)	7546 (22.9)
Skilled	154 (1.4)	790 (7.0)	395 (3.7)	1339 (4.1)
Other	187 (1.7)	147 (2.2)	706 (6.6)	1139 (3.5)
Education of mother				
No education	8595 (79.3)	7961 (69.8)	7105 (66.4)	23661 (71.8)
Primary	1801 (16.6)	3058 (26.8)	2864 (26.8)	7724 (23.4)
Secondary	400 (3.7)	237 (2.1)	490 (4.6)	1127 (3.4)
Higher	42 (0.4)	153 (1.3)	248 (2.3)	442 (1.3)
Age of mothers				
15–19	537 (5.0)	447 (3.9)	353 (3.3)	1337 (4.1)
20–24	2146 (19.8)	2247 (19.7)	2001 (18.7)	6395 (19.4)
25–29	3109 (28.7)	3685 (32.3)	3262 (30.5)	10,056 (30.5)
30–34	2270 (21.0)	2285 (20.0)	2443 (22.8)	6998 (21.2)
35–39	1666 (15.4)	1741 (15.3)	1713 (16.0)	5120 (15.5)
40–44	768 (7.1)	743 (6.5)	705 (6.6)	2217 (6.7)
45–49	341 (3.1)	260 (2.3)	230 (2.2)	830 (2.5)
Listening radio in the last week				
Not at all	7049 (65.1)	5728 (50.2)	7894 (73.7)	20671 (62.7)
Twice a week	2601 (24.0)	3658 (32.1)	1386 (12.9)	7645 (23.2)
Three times a week	326 (3.0)	2015 (17.7)	1428 (13.3)	3769 (11.4)
Almost everyday	860 (7.9)			860 (2.6)
Watching television in the last week				
Not at all	9931 (93.2)	7699 (67.6)	8814 (82.3)	26443 (80.3)
Twice a week	652 (6.1)	2,750 (24.1)	1,031 (9.6)	4433 (13.5)
Three times a week	69 (0.7)	944 (8.3)	863 (8.1)	1876 (5.7)
Toilet type				
Improved	574 (5.3)	1393 (12.4)	1044 (9.8)	3011 (9.2)
Unimproved	10,224 (94.7)	9878 (87.6)	9597 (90.2)	29699 (90.8)
Water source				
Improved	6156 (57.0)	5188 (46.1)	5994 (64.3)	17338 (55.3)
Unimproved	4639 (43.0)	6074 (53.9)	3325 (35.7)	14038 (44.7)

### Type of fuels used in the house and place of cooking food

**Fig. 1:**
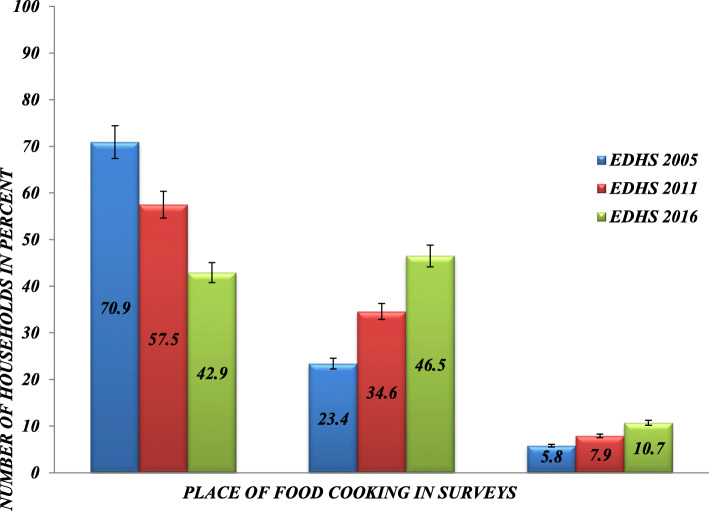
Household food cooking place, Ethiopian Demographic and Health Surveys, 2005-2016. The percent distribution is calculated by dividing the number of households reportedly cooking food inside the house, outside the house and in separate house to total number of households in each survey

The pooled data revealed that the proportion of households that used a solid biomass fuel as an energy source was 98.4% (95% CI = 98.1, 98.6) with 98.8% (95% CI = 98.4, 99.0), 99.5% (95% CI = 99.2, 99.7), and 96.9% (95% CI = 96.2, 97.4), respectively, in 2005, 2011, and 2016. The data concerning cooking foods’ locations indicate that 57%, 35%, and 8% cooked the foods inside the house, a separate building, and outside the house, respectively. The cooking location in each survey year shows that 71% of households cooked inside the house in 2005, and the number dropped to 43% in 2016 (Fig. 1). There was a declining trend in cooking food inside the house and an increasing trend of cooking in a separate house. Cooking the food outside the house increased from 5.8% in 2005 to 10.7% in 2016.

### Acute respiratory infection

Of the 30,895 children included in three surveys, 11.9% had an acute respiratory infection, 12.7% in 2005, 11.9% in 2011, and 11.1% in the 2016 survey. The results indicate a decline in the number of children with ARI from 2005 to 2011, although only slightly decreased from 2011 to 2016.

#### The distribution of ARI across variable categories

The prevalence of acute respiratory infection is 12.6% in the households that cooked the food inside the house. The prevalence of the disease is higher in the households that cooked the food outside the house and in a separate building with the respective percentage of 9.6% and 11.2%. The prevalence of ARI among families using solid biomass fuel is 11% compared with those who used non-solid fuel, which is about 6%. In addition, a low prevalence of infection was found in those mothers who reported ever breastfeeding, high paternal education status, and professional workers. Similarly, children of mothers with a high education status, between 44 and 49 years old reported a low ARI. Again, the mothers listening to the radio daily, watching television daily, and in the highest wealth quintiles also reported a lower ARI (Table 2).

**Table 2:** Acute respiratory infection on under-five children across different variable selected, EDHS 2005*–*2016

Variables	Category	Weighted frequency				ARI, weighted percentage (95% CI)			
		2005	2011	2016	Pooled	2005	2011	2016	Pooled
Breast feeding of child	Never	178	153	372	703	13.1 (6.1, 26.0)	8.7 (3.8, 18.5)	10.1 (5.0, 19.3)	10.5 (6.7, 16.2)
	Ever	9696	10545	9823	30064	12.7 (11.5, 14.0)	11.9 (10.8, 13.1)	11.2 (10.0, 12.5)	11.9 (11.3, 12.7)
Father’s education	No education	5750	5257	4636	15643	12.0 (10.6, 13.5)	12.3 (10.7, 14.0)	10.6 (9.0, 12.5)	11.7 (10.8, 12.6)
	Primary	3021	4474	3827	11322	14.0 (12.1, 16.1)	11.8 (10.2, 13.5)	12.9 (10.9, 15.2)	12.7 (11.6, 13.9)
	Secondary	992	514	748	2254	13.8 (11.0, 17.3)	10.3 (6.9, 15.0)	9.7 (6.4, 14.5)	11.6 (9.7, 13.9)
	Higher	107	332	411	851	3.3 (1.0, 10.5)	9.5 (5.2, 16.5)	3.7 (1.8, 7.3)	5.9 (3.8, 9.0)
Father’s occupation	Did not work	43	89	736	869	22.5 (9.6, 44.4)	19.7 (8.0, 40.7)	11.6 (7.9, 16.8)	13.0 (9.2, 17.8)
	Professional	166	311	391	868	6.2 (2.9, 12.5)	11.0 (6.2, 18.9)	4.4 (2.4, 7.9)	7.1 (4.8, 10.4)
	Sales	516	830	653	1999	7.6 (5.1, 11.2)	11.7 (8.5, 15.8)	11.0 (7.5, 0.160)	10.4 (8.4, 12.8)
	Agriculture	8674	8474	6384	23533	13.2 (11.9, 14.6)	12.1 (10.9, 13.4)	11.99 (10.3, 13.7)	12.5 (11.7, 13.3)
	Services	58	149	253	461	12.1 (5.0, 26.8)	14.8 (7.1, 0.283)	10.6 (6.2, 17.5)	12.1 (8.1, 17.8)
	Skilled	232	569	537	1338	12.7 (7.2, 21.4)	7.9 (5.0, 12.1)	11.5 (7.9, 16.6)	10.2 (7.8, 13.1)
	Unskilled	164	111	337	613	11.0 (6.6, 17.8)	12.5 (5.0, 27.9)	12.1 (8.2, 0.176)	11.9 (8.8, 15.9)
	Other	15	68	341	425	-	18.3 (8.8, 0.344)	5.8 (3.4, 9.6)	7.6 (4.8, 11.9)
Mother’s occupation	Did not work	7044	5009	5679	17732	11.8 (10.3, 13.4)	11.3 (9.9, 13.0)	10.3 (8.9, 11.9)	11.2 (10.2, 12.0)
	Sales	709	1762	1172	3643	16.1 (12.5, 20.4)	13.0 (10.7, 15.8)	12.8 (10.2, 16.0)	13.6 (11.9, 15.4)
	Agriculture	1880	2889	2305	7074	15.5 (12.9, 18.4)	12.6 (10.9, 14.6)	12.8 (10.4, 15.9)	13.5 (12,2, 14.2)
	Skilled	147	735	372	1254	9.6 (4.7, 18.7)	10.8 (7.7, 15.0)	11.7 (6.8, 19.6)	10.9 (8.3, 14.2)
	Other	174	240	668	1082	9.9 (5.6, 16.9)	(8.9 (5.1, 0.149)	8.7 (5.9, 12.6)	8.9 (6.8, 11.7)
Mother’s education	No education	7863	7446	6750	22059	12.6 (11.3, 14.1)	11.8 (10.5, 13.2)	11.1 (9.6, 12.7)	11.9 (11.1, 12.7)
	Primary	1679	2905	2734	7318	13.8 (11.3, 16.6)	12.4 (10.5, 14.5)	12.4 (10.4, 14.6)	12.7 (11.5, 14.0)
	Secondary	388	230	472	1090	10.4 (6.7, 15.9)	10.6 (6.1, 17.9)	8.2 (5.3, 12.5)	9.5 (7.3, 12.4)
	Higher	42	147	239	428	2.2 (0.6, 7.7)	8.4 (4.2, 16.2)	4.8 (2.3, 10.0)	5.8 (3.5, 9.5)
Household wealth quintile	Poorest	1904	1613	1510	5027	12.2 (10.0, 14.8)	11.7 (9.4, 14.4)	10.7 (8.3, 13.6)	11.6 (10.2, 13.1)
	Poorer	2206	3101	2551	7858	10.4 (8.6, 12.5)	12.0 (10.2, 14.0)	12.0 (9.7, 14.7)	11.5 (10.4, 12.8)
	Middle	2331	2553	2738	7622	14.6 (12.2, 17.4)	12.0 (9.9, 14.5)	11.8 (9.7, 14.4)	12.7 (11.4, 14.2)
	Higher	2422	2135	2296	6853	9.7 (12.7, 17.0)	11.4 (9.5, 13.7)	11.9 (9.7, 14.5)	12.7 (11.5, 14.1)
	Highest	1109	1327	1100	3536	11.7 (7.5, 12.5)	12.3 (9.5, 15.8)	6.5 (4.4, 9.4)	9.7 (8.2, 11.4)
Child sex	Male	5050	5531	5222	15803	12.8 (11.2, 14.5)	11.7 (10.3, 13.3)	11.0 (9.6, 12.5)	11.8 (11.0, 12.7)
	Female	4921	5198	4973	15092	12.6 (11.1, 14.3)	12.1 (10.8, 13.5)	11.3 (0.097, 13.1)	12.0 (11.1, 12.9)
Child age	O years	2208	2322	2243	6773	15.8 (13.3, 18.7)	14.7 (12.7, 17.0)	13.4 (11.2, 16.0)	14.6 (13.3, 16.1)
	1 years	1865	1875	1965	5705	14.7 (12.6, 17.1)	13.3 (11.1, 15.8)	13.8 (11.5, 16.5)	13.9 (12.6, 15.3)
	2 years	1865	2007	1892	5763	13.2 (11.1, 15.6)	12.0 (9.9, 14.4)	11.0 (8.8, 13.7)	12.1 (10.8, 13.5)
	3 years	2063	2321	1960	6344	10.9 (9.2, 12.9)	11.0 (9.1, 13.1)	10.3 (8.4, 12.5)	10.7 (9.6, 11.9)
	4 years	1970	2204	2135	6310	8.7 (7.1, 10.6)	8.6 (6.9, 10.6)	7.1 (5.5, 9.3)	8.1 (7.1, 9.3)
Age of mother	15–19	485	416	348	1249	13.0 (9.0, 18.4)	15.1 (9.9, 22.4)	13.6 (8.3, 21.3)	13.9 (10.9, 17.4)
	20–24	1959	2109	1887	5955	13.2 (10.7, 16.2)	11.7 (9.7, 14.1)	13.1 (11.0, 15.6)	12.6 (11.3, 14.1)
	25–29	2874	3442	3089	9405	13.1 (11.1, 15.4)	11.9 (10.2, 13.7)	9.8 (8.1, 11.8)	11.6 (10.5, 12.7)
	30–34	2081	2171	2350	6603	12.1 (10.1, 14.4)	11.0 (9.1, 13.2)	12.2 (9.9, 15.0)	11.7 (10.5, 13.1)
	35–39	1558	1637	1623	4818	12.5 (10.0, 15.5)	11.9 (9.8,14.5)	9.8 (7.7, 12.4)	11.4 (10.1, 12.9)
	40–44	709	714	675	2098	12.8 (9.4, 17.2)	14.6 (10.0, 20.8)	19.8 (6.9, 13.8)	12.4 (10.1, 15.2)
	44–49	306	240	223	768	10.7 (6.6, 16.8)	8.0 (4.7, 13.3)	11.8 (6.6, 20.2)	10.2 (7.5, 13.7)
Type of fuel used	Solid biomass fuel	9811	10553	9809	12995	12.8 (11.6, 14.1)	11.9 (10.8, 13.1)	11.2 (10.0, 12.5)	11.0 (11.3, 12.7)
	Non-solid fuel	130	53	326	508	4.7 (2.7, 8.0)	6.5 (1.3, 26.8)	6.8 (3.8, 12.0)	6.2 (3.9, 9.8)
Location of food preparation or cooking	Inside house	7050	6109	4328	17488	13.4 (11.9, 15.1)	12.2 (10.7, 13.9)	12.0 (10.4, 13.9)	12.6 (11.2, 13.6)
	Separate building	2343	3770	4770	10883	12.0 (10.1, 14.1)	11.0 (9.4, 12.7)	11.0 (9.4, 12.9)	11.2 (10.2, 12.3)
	Outside the house	575	843	1088	2506	6.8 (4.5, 10.2)	12.0 (10.0, 16.8)	8.3 (6.0, 11.4)	9.6 (7.9, 11.5)
Child size	Very large	2175	2028	1790	5992	14.9 (12.2, 18.1)	11.0 (9.1, 13.3)	14.1 (11.7, 16.9)	13.3 (11.9, 15.0)
	Larger than average	943	1317	1417	3677	10.1 (7.8, 12.8)	12.2 (9.9, 14.8)	7.7 (5.8, 10.2)	9.9 (8.6, 11.4)
	Average	4043	4184	4275	12503	11.7 (10.1, 13.5)	9.3 (8.0, 10.9)	9.7 (8.2, 11.5)	10.2 (9.3, 11.2)
	Smaller than average	724	964	1019	2708	8.9 (6.4, 12.1)	13.8 (10.7, 17.7)	11.1 (8.2, 14.8)	11.5 (9.7, 13.5)
	Very small	2071	2225	1619	5915	14.9 (12.7, 17.5)	16.5 (14.2, 19.0)	14.7 (12.2, 17.6)	15.4 (14.1, 16.9)
Toilet type	Unimproved	9394	9271	9117	2898	12.7 (11.5, 14.1)	12.2 (11.1, 13.5)	11.3 (10.1, 12.7)	9.6 (8.0, 11.5)
	Improved	546	1335	1018	27782	11.7 (8.5, 16.0)	9.6 (7.2, 12.6)	8.6 (6.2, 11.8)	12.1 (11.4, 12.9)
Drinking water source	Unimproved	5683	4926	3182	16301	13.4 (11.6, 15.4)	12.5 (11.0, 14.3)	11.4 (9.9, 13.2)	11.6 (10.7, 12.5)
	Improved	4255	5672	5692	13110	12.2 (10.7, 13.8)	11.1 (9.8, 12.6)	11.2 (9.2, 13.6)	12.5 (11.4, 13.6)
Residence	Urban	724	1372	1108	3204	8.8 (6.0, 12.9)	9.3 (7.2, 12.0)	7.0 (5.0, 9.6)	8.4 (7.0, 10.0)
	Rural	9247	9357	9087	27691	13.0 (11.0, 14.4)	12.3 (11.1, 13.5)	11.6 (10.3, 13.1)	12.3 (11.6, 13.1)
Stunting	Normal	2217	6201	5987	14404	11.7 (9.9, 13.8)	11.4 (10.0, 13.0)	10.9 (9.5, 12.4)	12.0 (11.1, 12.9)
	Stunted	2205	4388	3678	10272	13.8 (11.8, 16.1)	12.4 (11.0, 13.8)	12.0 (10.2, 14.0)	11.7 (10.7, 12.8)
Underweighting	Normal	2217	7734	7368	17318	11.7 (9.9, 13.8)	11.2 (10.1, 12.5)	10.8 (9.6, 12.1)	11.4 (10.6, 12.2)
	Underweighted	2205	2855	2327	7388	13.8 (11.8, 16.1)	14.0 (12.1, 16.1)	12.9 (10.6, 15.6)	13.0 (11.8, 14.3)
Wasting	Normal	2217	9620	8710	20547	11.7 (9.9, 13.8)	11.5 (10.4, 1.26)	10.9 (9.7, 12.2)	11.5 (10.7, 12.3)
	Wasted	2205	968	978	4151	13.8 (11.8, 16.1)	16.8 (13.7, 20.4)	14.8 (11.3, 19.1)	13.6 (12.0, 15.3)
Radio listening frequency in the last week of survey	Not at all	6486	5379	7549	19414	12.4 (110, 139)	11.9 (10.4, 13.5)	10.5 (9.2, 11.9)	11.5 (10.7, 12.4)
	Less than once a week	2375	3439	1299	7113	13.6 (11.4, 161)	11.7 (9.8, 13.9)	12.3 (9.5, 15.8)	12.4 (11.1, 13.9)
	At least once a week	305	1903	1347	3556	18.3 (12.1, 26.8)	12.2 (10.1, 14.6)	13.5 (10.6, 17.1)	13.2 (11.5, 15.2)
	Almost everyday	805	–	–	805	10.4 (7.5, 14.4)	–	–	10.4 (7.5, 14.4)
Television watching Frequency in the last week of survey	Not at all	9125	7910	8408	24742	12.9 (11.6, 14.4)	12.6 (11.2, 14.1)	11.1 (9.8, 12.6)	12.2 (11.4, 13.1)
	Less than once a week	611	2595	966	4172	11.8 (7.9, 17.3)	11.8 (9.8, 14.1)	14.0 (10.3, 18.7)	12.3 (10.6, 14.2)
	At least once a week	59	909	821	1789	9.7 (3.3, 25.0)	6.9 (4.6, 10.1)	7.9 (5.5, 11.1)	7.4 (5.7, 9.5)
	Almost everyday	160	–	–	160	3.5 (1.9, 6.6)	–	–	3.5 (1.9, 6.6)

Note: No education: those who did not attend school, Primary: those who completed grades 1–8, Secondary: those who completed grades 9–12, Higher: those with a college certificate, diploma, or above; Improved water: household’s drinking water source is from protected spring, protected well, and piped water, Unimproved water source: household’s drinking water source is from unprotected spring, unprotected well, tanker truck, surface water, irrigation channel, and cart with small truck; Improved toilet: pit latrine with slab; VIP, flush to septic tank, flush to sewer system, and flush to pit latrine compost; Unimproved toilet: pit latrine without slab, bucket latrine, hanging latrine, no-facility, or open field; occupation of mother and house head is based on EDHS category

#### The association of ARI with place of cooking food in the house

The empty model indicates a variation in the risk of having ARI among children in households and surveys with respect to random intercepts of 0.13 and 0.37. In model-II, where the survey-level variables (place of food cooking) were used, families cooking the food outdoor had a 30% (95% CI = 0.56, 0.88) lower risk of having under-five children with ARI compared to those cooking inside the home. The random intercepts for the households and survey in model-II also indicate significant differences among households within the survey (between households of the same study) and surveys having under-five children with ARI. The full model (after including all the variables in the models) showed that cooking the food outdoor had a 44% (AOR = 0.56, 95% CI = 0.40, 0.75) lower risk of having under-five children with ARI compared to cooking inside the house. Similarly, the children’s risk of having ARI among households that cooked the food in a separate kitchen was 19% less likely (AOR = 0.81, 95% CI = 0.69, 0.96). The standard deviations of random intercepts and random slopes in a full model were higher than their respective standard errors showing variability from survey to survey. However, the standard deviation of the household’s random intercepts was near zero, meaning there was no house to house variation in this term (Table 3).

**Table 3:** Multilevel logistic regression on the association of food cooking places with ARI in under-five children in controlling other variables, EDHS, 2005–2016

Variables	Category	Model-I OR(95% CI)	Model-II OR (95% CI)	Model-III OR (95% CI)
Child age	0 years			1.00
	1 years			0.90 (0.74, 1.09)
	2 years			0.73 (0.60, 0.88)
	3 years			0.66 (0.55, 0.79)
	4 years			0.48 (0.39, 0.59)
Child size	Very large			1.00
	Larger than average			0.75 (0.58, 0.96)
	Average			0.73 (0.60, 0.89)
	Smaller than average			0.85 (0.65, 1.11)
	Very small			1.16 (0.94, 1.43)
Stunting	Stunted			1.00
	Normal			1.13 (0.94, 1.35)
Underweighting	Underweight			1.00
	Normal			0.77 (0.61, 0.98)
Wasting	Wasted			1.00
	Normal			1.00 (0.81, 1.23)
Education of father	No education			1.00
	Primary			1.10 (0.92, 1.31)
	Secondary			1.02 (0.73, 1.41)
	Higher			0.95 (0.53, 1.80)
Occupation of father	Did not work			1.00
	Professional			0.64 (0.36, 1.13)
	Sales			0.84 (0.51, 1.38)
	Agriculture			0.92 (0.59, 1.44)
	Services			1.15 (0.56, 2.36)
	Skilled			0.89 (0.48, 1.53)
	Unskilled			1.00 (0.50, 1.59)
	Other			0.51 (0.26, 1.02)
Occupation of mother	Did not work			1.00
	Sales			1.22 (0.97, 1.52)
	Agriculture			1.17 (0.97, 1.41)
	Skilled			1.00 (0.71, 1.41)
	Other			0.69 (0.45, 1.04)
Education of mother	No education			1.00
	Primary			1.09 (0.91, 1.30)
	Secondary			0.98 (0.62, 1.56)
	Higher			1.42 (0.67, 3.00)
Quintile	Poorest			1.00
	Poorer			0.93 (0.73, 1.19)
	Middle			0.96 (0.74, 1.23)
	Higher			0.98 (0.76, 1.25)
	Highest			0.75 (0.56, 1.01)
Frequency of listening radio the last week	Not at all			1.00
	Less than once a week			1.23 (1.01, 1.50)
	At least once a week			1.50 (1.19, 1.88)
	Almost everyday			0.96 (0.54, 1.70)
Frequency of watching television in the last week of survey	Not at all			1.00
	Less than once a week			0.84 (0.67, 1.06)
	At least once a week			0.59 (0.38, 0.93)
	Almost everyday			0.26 (0.05, 1.34)
Age of mother	15-19			1.00
	20-24			0.94 (0.62, 1.41)
	25-29			0.91 (0.61, 1.36)
	30-34			0.96 (0.64, 1.44)
	35-39			0.87 (0.57, 1.34)
	40-44			1.07 (0.65, 1.78)
	44-49			0.64 (0.36, 1.15)
Toilet type	Unimproved			1.00
	Improved			0.96 (0.71, 1.29)
Drinking water source	Unimproved			1.00
	Improved			1.02 (0.86, 1.21)
Residence	Rural			1.00
	Urban			0.77(0.53, 1.14)
Food cooking place	Inside the house		1.00	1.00
	In separate building		0.86 (0.75, 0.99)	0.81(0.69, 0.96)
	Outside the house		0.70 (0.56, 0.88)	0.56 (0.40, 0.75)
Random intercept for households within survey		0.13^a^	0.10^a^	0 .003
Random intercept for survey		0.37^a^	0.29^a^	0.39^a^
Random slope for place of cooking			.08^a^	0.11^a^

^a^The denotes the statistical significance of random intercept and slope

## Discussion

The three surveys show that more than 98% of the households use solid biomass fuel for cooking food even if these fuels are known to be emitting a large number of indoor air pollutants which implies that the children’s exposure to biomass and charcoal fuel leads to a high chance of developing ARI [40]. This study found only 9.6% of the total households prepared food outside the living quarters, which was different from other studies conducted in African countries that reported 18% in East Africa and 43% in West Africa [41].

The current finding shows a lower risk of having an ARI in children under-five in households that cook the food outside the home compared to those cooking inside the house (after controlling for other variables). This finding is consistent with a prior study conducted in 27 African countries [41] and Afghanistan [15]. This indicates that cooking food in a separate building will lower the risk of ARI in children compared to cooking inside a building; this complies with findings from Tanzania [42], Nigeria [43], and the most recent one from India [18], all based on demographic and health survey data.

The older children have a lower ARI risk than children below 1 year, suggesting less exposure to induced indoor air pollutants due to less time spent indoors. Mothers with younger children prefer to cook indoors while caring for their children. The finding is similar to previous studies in urban areas of the Oromia region, Ethiopia [33]; in Wondo-Genet district, southern Ethiopia [23]; and in Afghanistan [34].

The mother’s watching of television at least once a week was protective of her child to develop ARI, which is consistent with the prior study [32]. Watching television may increase awareness about the risks of solid biomass fuel, cooking place, and disseminate knowledge about the programs and policies related to their child’s healthcare services.

The variation in the number of children with ARI among households of different surveys from multilevel modeling suggests a change in the practice of families using non-solid biomass or preparing their food outside and/or ventilated kitchen. This could result from a country-level effort to improve the use of non-solid biomass fuels, and improved stove use, by health extension workers since 2003 [44–48].

The higher risk of having children under-five with ARI in the households while cooking inside compared to both the outdoor environment and separate buildings implies that exposures to a high concentration of particulate matter (PM10) and NO_2_ are predisposing factors for ARI, as reported in a prior study in Tanzania [42], and poor ventilation of the house [40]. Thus, children in households that cook food inside the homes were exposed to the pollutants, resulting in lung function impairment. A prior finding revealed a significant reduction in forced expiratory lung volumes in biomass fuel users [42]. The histopathological finding in under-five children who spent, on average, 4–5 h a day inside the kitchen revealed a detailed necroscopy [43]. Biomass smoke exposure increased forced expiratory volume (FEV)1/force vital capacity (FVC), indicating a significant loss of vital capacity sufficiently high to be responsible for both obstructive and restrictive pulmonary diseases in long exposure [44]. As the duration and the intensity of exposure increase, the probability of having altered pulmonary function test results is higher [26].

The problem can be reduced by changing the country’s community practices, mainly shifting to alternative energy sources, using an improved stove, and behavior change. These were indicated to be effective [49, 50]. A prior study revealed improved stoves that effectively reduce the pollutants’ emission in the country [51], although the use is low [52]. Therefore, effort on the wide-scale implementation of Climate Resilient Green Economy (CRGE) with the dissemination of improved stoves as one of the strategies remains essential to improve the coverage and use to minimize the problem [53].

## Strengths and limitations of the study

This study’s strengths include that it used nationally representative data with a representative sample size collected in three different periods and analyzed using multilevel modeling. However, it has the following limitations: the data were based on a cross-sectional study design. The variables like ARI and size of the children were based on the respondent’s self-report, leading to bias.

## Conclusion

More than 10% of children suffer from an acute respiratory infection, and about 98% of households used solid biomass fuel in the country. In the families where the food was cooked outdoor, there was a lower risk of having children with ARI than that cooked inside the house. There was an inclining trend in the number of households that cooked the food in the outdoor environments and a separate building. The multilevel modeling shows a variation in the infection of the disease among households of different surveys. But still, an effort remains mainly on awareness creation on cooking indoor homes of well-ventilated and shifting to alternative energy sources. Besides, promoting the use of improved stoves can affect reducing the problem in the country. This can be through the integration of health sectors, mainly health extension programs of the Ministry of Health, designed to deliver health services at the grass-root level, including healthy housing, and the Ministry of Water, Irrigation, and Energy providing alternative energy sources, mainly biogas and others.

## Data Availability

The datasets used and/or analyzed during the current study belong to DHS program. The authors can provide in discussion with the data owner.

## Abbreviations

ARI: Acute respiratory infection FEV Forced expiratory volume FVC Force vital capacity OR Odds ratio DHS Demographic Health Survey PM10 Particulate matter
